# Supplementary material for: Restrained Eating Is Associated with Lower Cortical Thickness in the Inferior Frontal Gyrus in Adolescents
Source: Brain Sci. 2021 Jul 23;11(8):978. doi: 10.3390/brainsci11080978 (PMC8394556; doi:10.3390/brainsci11080978)
Supplement: Supplementary file 1 [file brainsci-11-00978-s001.zip › brainsci-1287876-supplementary.pdf]

Supplementary materials

Table S1. Executive function performance in adolescents

Variables	Uncontrolled eating (n=35)	Restrained eating (n=29)	Low problematic eating (n=44)	F	p
Wisconsin Card Sorting Test – total errors	37.49 (22.43)	36.93 (20.54)	28.05 (15.06)	0.223	0.114
Trail Making Test – B minus A	46.18 (30.25)	42.41 (18.35)	41.33 (19.29)	0.427	0.654
Letter-Number Sequencing (WAIS)	17.29 (4.83)	17.59 (4.39)	18.17 (3.56)	0.134	0.875
Stroop – interference score	2.90 (6.33)	3.65 (6.71)	2.86 (6.17)	0.039	0.962
Continuous performance test II – commission errors	23.67 (5.99)	23.19 (7.98)	23.19 (6.08)	1.502	0.228

Table S2. Description of eating patterns, demographical variables, and clinical characteristics of the three clusters in the neuroimaging subsample of adolescents.

**Domains**	**Variables**	**Uncontrolled eating (n=19)**	**Restrained eating (n=16)**	**Low problematic eating (n=26)**	**F**	**p**
Intracluster variables (TFEQ; centered)	Cognitive restraint	0.02 (0.91)	1.07 (0.63)	-0.80 (0.58)	33.08	**<0.001**
	Disinhibited eating	1.00 (0.74)	-0.71 (0.80)	-0.29 (0.72)	25.82	**<0.001**
	Emotional eating	1.07 (0.86)	-0.44 (0.59)	-0.72 (0.38)	49.63	**<0.001**
Extracluster validation (BITE)	BITE symptoms	6.64 (4.72)	3.07 (2.37)	2.35 (1.90)	10.77	**0.001**
Demographic and clinical variables	Age	14.53 (1.93)	14.33 (1.59)	14.23 (1.56)	0.17	0.845
	Sex	11 females (58%)<br>8 males (42%)	7 females (47%)<br>15 males (53%)	13 females (50%)<br>13 males (50%)	$X^2(2)=0.474$	0.789
	Waist (cm) / Height (cm)	0.52 (0.09)	0.55 (0.08)	0.47 (0.08)	5.000	0.009
	BMI	27.69 (6.12)	28.70 (5.61)	22.97 (5.61)	5.946	0.005
	Body weight status	6 lean (32%)<br>4 overweight (21%)<br>9 obese (47%)	3 lean (20%)<br>0 overweight (0%)<br>12 obese (80%)	16 lean (62%)<br>4 overweight (15%)<br>6 obese (23%)	$X^2=17.57$	**0.002**
	Anxiety and depression (HADS)	9.21 (4.49)	6.80 (4.38)	6.23 (4.39)	2.63	0.081

Table S3. Executive function performance in adults

Variables	Uncontrolled eating (n=54)	Restrained eating (n=61)	Low problematic eating (n=60)	F	p
Wisconsin Card Sorting Test – total errors	29.61 (17.60)	28.39 (18.55)	25.85 (12.94)	0.139	0.870
Trail Making Test – B minus A	41.59 (25.02)	37.51 (25.22)	37.17 (21.03)	0.010	0.990
Letter-Number Sequencing (WAIS)	10.76 (2.33)	11.28 (2.44)	11.32 (2.04)	0.653	0.522
Stroop – interference score	3.82 (5.89)	2.584 (7.34)	3.73 (7.13)	0.976	0.379
Continuous performance test II – commission errors	12.37 (6.70)	12.64 (5.91)	13.00 (6.87)	0.146	0.864

Table S4. Description of eating patterns, demographical variables, and clinical characteristics of the three clusters in the neuroimaging subsample of adults.

Domains	Variables	Uncontrolled eating (n=32)	Restrained eating (n=42)	Low problematic eating (n=32)	F	p
Intracluster variables (TFEQ; centered)	Cognitive restraint	0.46 (0.86)	0.55 (0.64)	-1.01 (0.51)	56.10	**<0.001**
	Disinhibited eating	0.95 (0.73)	-0.19 (0.44)	-0.75 (0.60)	69.38	**<0.001**
	Emotional eating	1.16 (0.54)	-0.17 (0.49)	-0.91 (0.37)	160.0	**<0.001**
Extracluster validation (BITE)	BITE symptoms	9.50 (4.41)	4.55 (3.22)	284 (2.40)	33.11	**<0.001**
Demographic and clinical variables	Age	31.31 (7.49)	30.50 (9.66)	31.53 (7.50)	0.157	0.855
	Sex	21 females (66%)<br>11 males (34%)	26 females (62%)<br>16 males (38%)	11 females (34%)<br>21 males (66%)	$X^2(2)=7.757$	**0.020**
	Waist (cm) / Height (cm)	0.59 (0.10)	0.56 (0.10)	0.49 (0.08)	8.63	**<0.001**
	BMI	30.39 (6.22)	29.59 (6.55)	25.07 (5.28)	7.28	**0.001**
	Body weight status	8 lean (25%)<br>4 overweight (12.5%)<br>20 obese (62.5%)	9 lean (21%)<br>20 overweight (48%)<br>13 obese (31%)	22 lean (69%)<br>7 overweight (22%)<br>3 obese (9%)	$X^2(2)=34.09$	**<0.001**
	Anxiety and Depression (HADS)	7.09 (4.43)	5.43 (3.42)	5.66 (4.32)	1.724	0.184

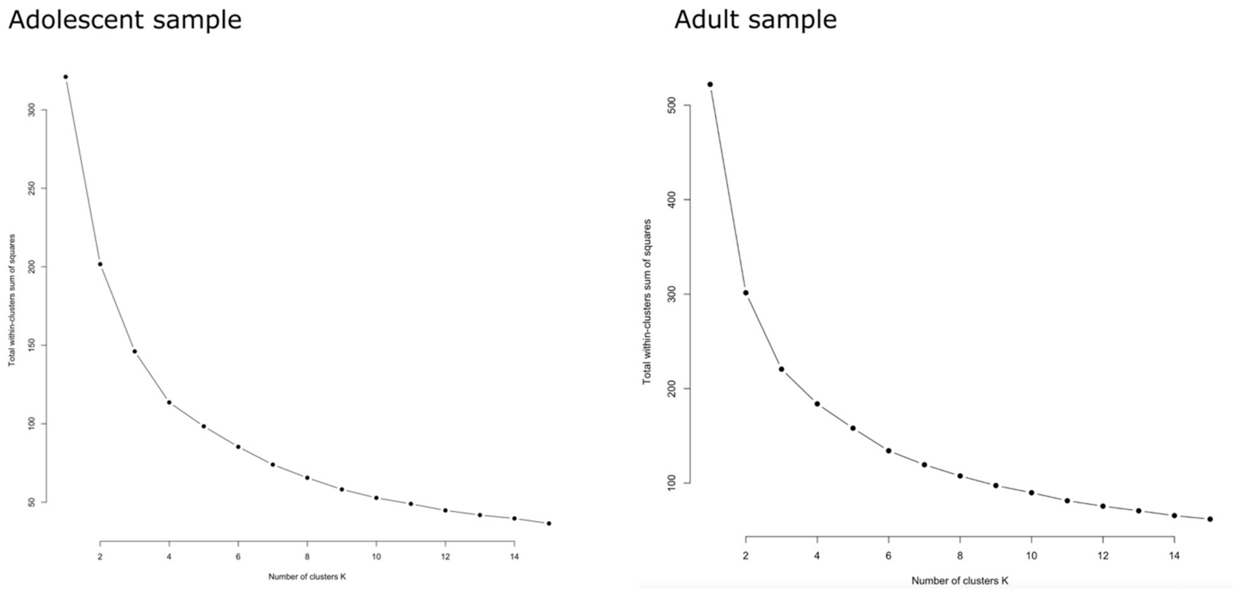

Figure S1. Within clusters sum of squares in the adolescent and the adult samples
